# Study protocol: exercise training for treating major depressive disorder in multiple sclerosis

**DOI:** 10.1186/s12883-024-03634-y

**Published:** 2024-04-17

**Authors:** Robert W. Motl, Charles H. Bombardier, Jennifer Duffecy, Brooks Hibner, Alison Wathen, Michael Carrithers, Gary Cutter

**Affiliations:** 1 Department of Kinesiology and Nutrition, University of Illinois at Chicago, Chicago, IL 60612 USA; 2 Department of Rehabilitation Medicine, University of Washington, Seattle, WA USA; 3 Department of Psychiatry, University of Illinois at Chicago, Chicago, IL USA; 4 Department of Neurology and Rehabilitation, University of Illinois at Chicago, Chicago, IL USA; 5 Department of Biostatistics, University of Alabama at Birmingham, Birmingham, AL USA

**Keywords:** Exercise, Physical activity, Depression, Mood, Mental health, Behavior change, Multiple sclerosis, Neurological disease, Telehealth

## Abstract

**Background:**

Major depressive disorder (MDD) is prevalent, yet sub-optimally treated among persons with multiple sclerosis (MS). We propose that exercise training may be a promising approach for treating depression in persons with MS who have MDD. Our primary hypothesis predicts a reduction in depression severity immediately after an exercise training intervention compared with minimal change in an attention control condition, and the reduction will be maintained during a follow-up period.

**Methods:**

This study involves a parallel-group, assessor-blinded RCT that examines the effect of a 4-month home-based exercise training intervention on depression severity in a sample of persons with MS who have MDD based on the MINI International Neuropsychiatric Interview. The primary outcomes of depression severity are the Patient Health Questionnaire-9 and Hamilton Depression Rating Scale. Participants (*N* = 146) will be recruited from within 200 miles of the University of Illinois at Chicago and randomized (1:1) into either a home-based exercise training condition or control condition with concealed allocation. The exercise training and social-contact, attention control (i.e., stretching) conditions will be delivered remotely over a 4-month period and supported through eight, 1:1 Zoom-based behavioral coaching sessions guided by social-cognitive theory and conducted by persons who are uninvolved in screening, recruitment, random assignment, and outcome assessment. We will collect outcome data at 0, 4 and 8 months using treatment-blinded assessors, and data analyses will involve intent-to-treat principles.

**Discussion:**

If successful, the proposed study will provide the first Class I evidence supporting a home-based exercise training program for treating MDD in persons with MS. This is critical as exercise training would likely have positive secondary effects on symptoms, cognition, and quality of life, and provide a powerful, behavioral approach for managing the many negative outcomes of MDD in MS. The program in the proposed research is accessible and scalable for broad treatment of depression in MS, and provides the potential for integration in the clinical management of MS.

**Trial registration:**

The trial was registered on September 10, 2021 at clinicaltrials.gov with the identifier NCT05051618. The registration occurred before we initiated recruitment on June 2, 2023

**Supplementary Information:**

The online version contains supplementary material available at 10.1186/s12883-024-03634-y.

## Introduction

Multiple sclerosis (MS) is an immune-mediated, neurodegenerative disease of the central nervous system (CNS). There are an estimated one million adults living with MS in the United States [1]. This disease is characterized by demyelination and transection of axons and loss of neurons in the CNS [2]. The extent and location of CNS damage results in consequences including motor and cognitive dysfunction, fatigue, and major depressive disorder (MDD) [3].

MDD is characterized by persistently depressed mood or loss of interest in usual activities plus the presence of at least 5 of 9 symptoms that cause significant impairment in daily life [4]. The prevalence of MDD in persons with MS is nearly 1.7 times higher than the general population [5]. One recent systematic review reported the prevalence of MDD among persons with MS as 23.7% [6] and this translates into an estimated 250,000 people living with MS and MDD in the United States.

MDD has widespread, negative effects on the lives of people with MS [3]. The presence of MDD is associated with worsening of other symptoms such as fatigue, poorer neuropsychological functioning, and lower health-related quality of life (HRQOL) [3].

The prevalence and burden of MDD in MS underscore the critical importance of efficacious antidepressant treatments, yet such treatments are sorely lacking in MS. For example, the American Academy of Neurology concluded that there is insufficient evidence from randomized controlled trials (RCTs) for recommending antidepressants for treating MDD in MS [7]. One meta-analysis [8] of RCTs concluded that “CBT can be an effective intervention for reducing moderate depression, over the short term in patients with MS.” Yet, nearly 50% of participants do not benefit from CBT [9].

Exercise training is a promising therapy for improving depressive symptomology and managing MDD in MS [10]. Exercise training has yielded a moderate-to-large antidepressant effect in persons from the general population who have MDD [11–14]. Exercise training further has improved depressive symptomology in MS [15–17], and those meta-analyses offer critical insights for informing the exercise training parameters for treating MDD. The first meta-analysis indicated that both aerobic and resistance exercise training can yield a reduction in depressive symptoms for people with MS [15]. The second meta-analysis quantified the effect of exercise on depression in adults with neurologic disorders, including MS [16], and noted that interventions meeting physical activity guidelines yielded a reduction in depression that was two-times larger than interventions that did not meet physical activity guidelines. The third meta-analysis examined variables that moderate the effects of exercise on depressive symptoms among people with MS [17], and there was a dose–response effect for frequency (days/week) of exercise on reductions in depressive symptoms with the largest effect occurring for three days/week of exercise training.

The aforementioned meta-analyses identified four major limitations of previous research on exercise training for treating depression in MS [15–17]. The most pressing limitation is that the samples of persons with MS were not pre-screened for MDD [15–17]. Another limitation is that the exercise training programs were administered in supervised, center-based settings that present barriers associated with accessibility (e.g., distance, transportation, and costs) that likely influence adoption and maintenance of exercise behavior. An additional limitation is the lack of standardization of the exercise training prescription included in RCTs. The final limitation is the lack of follow-up regarding the durability of changes in depressive symptoms following exercise training.

We designed a RCT that is based on sound scientific rationale established through critical review and analysis of the relevant literature [10, 15–17], and further capitalizes on our experiences with home-based delivery of exercise training programs in MS [18–22]. To that end, we propose a parallel group, RCT for examining the efficacy of a home-based, exercise training program informed by prescriptive guidelines [23, 24] and guided by social cognitive theory (SCT)-based remote behavior coaching compared with a social-contact, attention control condition (i.e., stretching) for yielding immediate and sustained reductions in the severity of depressive symptoms among persons with MS who have MDD.

## Methods/design

There is only one protocol version and it will follow the Standard Protocol Items: Recommendations for Interventional Trials (SPIRIT) guidelines.

### Aims, design, and setting of the study

The primary aim examines the efficacy of a 4-month, home-based aerobic and resistance exercise training intervention compared with a 4-month, home-based stretching and flexibility intervention (i.e., social contact, attention control condition) for immediate and sustained (i.e., 4-months post-intervention) reductions in depression severity among persons with MS who have impaired MDD.

The secondary aim examines the efficacy of the exercise training intervention compared with the control condition for immediate and sustained improvements in fatigue, cognition, and HRQOL among persons with MS who have impaired MDD.

The tertiary aim involves a manipulation check and examines the efficacy of the exercise training intervention compared with control condition for immediate and sustained improvements in exercise behavior, physical activity, aerobic fitness, and muscle strength for persons with MS who have impaired MDD.

The study aims will be tested using a parallel-group, assessor-blinded RCT design. This study does not include a data safety monitoring board, but there is a data safety monitoring plan and safety monitor for oversight.

### Participants

#### Recruitment

We will recruit participants residing within 200 miles of the University of Illinois Chicago (UIC) campus located in Chicago, IL USA through distribution of study materials (flyers, business and post cards, and advertisements) among the Greater Illinois and other regional Chapters of the National MS Society; North American Research Committee on Multiple Sclerosis and iCONQUER MS Registries; waiting rooms of 10 + local MS Centers and Neurology offices; local community centers, churches, libraries, and physical therapy clinics; community events and MS support group meetings; focal study website (https://metsforms.ahs.uic.edu); UIC and professional listservs; and social media.

##### Inclusion/exclusion

We will assess inclusion/exclusion during a scripted phone screening by the project coordinator, and this will involve a two-stage process with the inclusion and exclusion criteria listed in Table 1.

**Table 1:** Inclusion and exclusion criteria by screen

Inclusion criteria	Exclusion criteria
Screen 1: First Level Criteria	
Physician-confirmed diagnosis of multiple sclerosis	High risk for contraindications of possible injury or death when undertaking strenuous or maximal exercise using the Physical Activity Readiness Questionnaire [25]
Relapse and steroid free in the past 30 days	Severe cognitive impairment based on Modified Telephone Interview for Cognitive Status score of less than 18 [26]
Internet and email access	Current high suicidal risk based on the Columbia-Suicide Severity Rating Scale screening version-recent [27]
Willingness to complete the testing and questionnaires, wear the accelerometer, undergo randomization, and engage in exercise training	
Insufficient physical activity based on a Health Contribution Score of less than 14 units from the Godin Leisure-Time Exercise Questionnaire [28]	
Ability to ambulate without assistance and Patient-Determined Disease Steps score between 0 and 2 (i.e., mild ambulatory disability) [29]	
Age between 18 and 64 years	
English as a primary language	
Not pregnant and/or nursing	
Presence of mild or more severe depressive symptoms based on Beck Depression Inventory – Fast Screen score of 4 or greater [30, 31]	
Screen 2: Second Level Criteria	
Major Depressive Disorder based on the MINI International Neuropsychiatric Interview [32]	Other severe mental illness (obsessive–compulsive disorder, schizophrenia, bipolar or other psychotic disorders, or recent or current addiction) as indicated by the MINI [32]

#### Attrition

We have experienced low attrition (10%) in our previous RCTs of the exercise training protocol in this proposal [19], and we note recent data from a meta-analysis suggesting an attrition rate of ~ 10% across 40 RCTs of exercise training in MS [33]. We believe attrition could be higher in this RCT based on depressive symptomology in MDD resulting in poor motivation and adherence for exercise engagement. We conservatively planned for a higher attrition rate of 20% for the proposed RCT, and recognize that retention might be a challenge, although we are including SCT-based content and 1:1 zoom-based behavioral coaching for maximizing retention and adherence [34] as is appropriate for persons with MS who have elevated depressive symptoms [35].

#### Power analysis and sample size

The power analysis was conducted in G*Power, Version 3.1 using F test for Test family and ANOVA: Repeated measures, within-between interaction for Statistical test. We estimated the sample necessary for detecting a Condition (2 levels of between-subjects factor: Intervention vs. Control) × Time (2 levels of within-subjects factor: 0 and 4 months) interaction on the primary outcomes of depression severity (i.e., 9-item Patient Health Questionnaire (PHQ-9; [36]) and Hamilton Depression Rating Scale (HDRS-17; [37]). We did not include 3 time-points as this assumes linear change across all 3 time points in G*Power 3.1, and we expected change between 0 and 4 months for the intervention condition, followed by stability between 4 and 8 months. The effect size (Cohen’s *f* = 0.18) was from our previous meta-analyses [15] regarding the effect of exercise training on depressive symptoms in persons with MS. The power analysis included assumptions of reliability for the within-subjects factor of ICC = 0.50, two-tailed *α* = 0.025, and *β* = 0.05 (i.e., 95% power); the *α* = 0.025 was selected based on two primary outcomes. The power analysis indicated the minimal total sample size for testing the Time × Condition interaction of 122 participants (61 per group), and we anticipate a dropout rate of ~ 20% resulting in a projected recruitment of 146 participants.

### Outcomes

#### Overview

The primary outcomes are the PHQ-9 [36] and HDRS-17 [37] as measures of depression symptom severity appropriate for MDD, whereas the secondary outcomes include measures of fatigue, cognitive performance, and HRQOL. The tertiary outcomes are exercise behavior, accelerometry as a device-based measure of free-living PA, and aerobic and muscle fitness. All outcomes will be assessed at baseline (0 months), immediate follow-up (4 months), and long-term follow-up (8 months) by treatment-blinded assessors. The assessors will not be involved in random assignment or delivery of the conditions, and will not directly communicate with the behavior coaches about participants. Participants themselves will be instructed not to discuss exercise routines with assessors, and why it might bias the evaluators.

#### Primary outcome measures

We include two outcomes for depression severity, as one is self-reported (primary) and the other is a semi-structured, interviewer-rated measure (secondary). The logic is that the self-report change in depression severity should be confirmed with the semi-structured, interviewer-rated change, as change in the former is more likely, but could represent a self-report bias associated with participants not being blinded regarding treatment condition.

#### Self-reported depression severity

The PHQ-9 is a brief, patient-reported depression severity measure [36]. The PHQ-9 is unidimensional [38], has good test–retest reliability [39], and has validated thresholds of mild, moderate, moderately severe and severe depression [36]. The PHQ-9 accurately discriminates differential treatment response among groups independently judged to have persistent MDD, partial remission, and full remission [39]. The PHQ-9 has a valid threshold for determining depression remission (less than 5) [39], and an established threshold for minimal clinically significant difference for individual change (5 points on 0–27 point scale) [39].

#### Interviewer-rated depression severity

The 6-item Maier subscale [40] of the HDRS-17 [37] is a semi-structured, interviewer-rated measure that is administered by treatment-blinded assessors. The Maier subscale was developed using Rasch analyses and provides a unidimensional subscale that has equivalent or greater sensitivity to treatment effects compared with the HDRS-17 [41, 42]. The Maier subscale has been recommended specifically for depression treatment trials in patients with medical comorbidities because the measure includes no somatic items [42]. The Maier has a valid cutoff for remission (4 or less) [42].

#### Secondary outcome measures

We will include the secondary end-points of fatigue [43], cognitive performance [44], and HRQOL [45], as changes in depression are often accompanied by changes in fatigue, neuropsychological function, and HRQOL [3]. These outcomes will anchor depression changes with other clinical end-points of substantial relevance for persons with MS who have MDD.

#### Fatigue

The perception of fatigue severity will be measured using the Fatigue Severity Scale (FSS) [43]. The FSS has 9 items rated on a 7-point scale regarding the severity of fatigue symptoms during the past 7 days. The item scores are averaged into a measure of fatigue severity that ranges between 1 and 7. FSS scores of 4 or above are indicative of severe MS-related fatigue [43], and the MDC for the FSS is 1.9 points [46]. There is evidence for the internal consistency, test–retest reliability, and validity of FSS scores as a measure of fatigue severity in MS [43].

#### Cognitive performance

Cognitive performance is a secondary outcome that will be assessed using the Brief International Cognitive Assessment for MS (BICAMS) [44]. The BICAMS battery includes the Symbol Digit Modalities Test (SDMT), first five learning trials of the California Verbal Learning Test-II (CVLT-II), and first three learning trials of the Brief Visuospatial Memory Test-Revised (BVMT-R) for measuring information processing speed, verbal learning and memory, and visuospatial learning and memory, respectively [44, 47]. The SDMT involves pairing 9 abstract geometric symbols with single digit numbers in a key, and orally stating the correct numbers for unpaired symbols as rapidly as possible for 90 s. The primary outcome of the SDMT is the number of correct responses provided in 90 s (i.e., raw score). The CVLT-II involves an examiner reading aloud a list of 16 words (four items belonging to four categories such as vegetables, animals, furniture, modes of transportation) that are randomly arranged; this is done five times in the same order at a rate of approximately one word per second. Participants recall as many items as possible, in any order, following each reading of list. The primary outcome of the CVLT-II is the total number of correct words identified over the five trials (i.e., raw score). The BVMT-R involves three trials of the examiner presenting a 2 × 3 array of abstract geometric figures approximately 15 inches in front of the participant for 10 s. The array is then removed and participants draw the array as precisely as possible with the figures in the correct location. Each drawing is scored based on accurately portraying each figure and its correct location using a 0–2 scale. The primary outcome of the BVMT-R is the total raw score across the three trials. There are benchmark scores for the cognitive tests included in the BICAMS that are associated with specific degrees of impairment in work status [48].

#### HRQOL

The 29-item Multiple Sclerosis Impact Scale (MSIS-29) [45] provides a disease-specific measure of physical (20 items) and psychological (nine items) HRQOL. The scores range between 0 and 100 with lower MSIS-29 scores representing higher HRQOL. There is evidence for the reliability and validity of the MSIS-29 in samples of persons with MS [45, 49].

#### Tertiary outcome measures

We will measure change in exercise behavior using the Godin Leisure-Time Exercise Questionnaire (GLTEQ) [50] and minutes/day of moderate-to-vigorous physical activity (MVPA) from accelerometry as a measure of free-living physical activity. We will measure aerobic capacity and muscle strength using accepted measures and protocols in MS [51]; this permits an additional check on the manipulation of performing the GEMS exercise-training protocol.

#### Self-reported exercise behavior

The GLTEQ measures the frequency of strenuous, moderate, and mild physical activity performed for periods of 15 min or more over a 7-day period [50, 52], and it will be scored as the Health Contribution Score (HCS) [28]. The HCS only includes strenuous and moderate physical activity. The HCS is computed by multiplying the frequencies of strenuous and moderate activities by 9 and 5 METs, respectively, and then summing the weighted scores. The HCS can be converted into one of three categories, namely, insufficiently active (i.e., score < 14 units), moderately active (i.e., score between 14 and 23 units), and active (i.e., score ≥ 24 units).

#### Device-measured free-living physical activity

The ActiGraph model GT3X + accelerometer (Actigraph Corporation, FL) worn during a seven-day period will provide a measure of free-living physical activity as minutes/day of MVPA. The ActiGraph accelerometer will be placed on an elastic belt that is worn snuggly around the waist over the non-dominant hip during the waking hours of a seven-day period. The data from the ActiGraph accelerometer will be downloaded and processed using the low frequency extension (i.e., filter for increasing the devices sensitivity) into one-minute epochs using ActiLife software (Actigraph Corporation, FL), and then scored for wear time and minutes/day of MVPA using MS-specific cut-points [53]. Only data from valid days (wear time ≥ 600 min) will be included in the analyses [53] and this will be confirmed with the compliance log. We will average data over two or more valid days for the outcome of minutes/day of MVPA, as this provides a reliable estimate of free-living physical activity behavior over a seven-day period [53]. Other measures such as steps/day and minutes/day spent in light physical activity and sedentary behavior [54] can be generated as additional end-points for understanding change in free-living physical activity.

#### Aerobic capacity

Cardiorespiratory fitness will be operationalized as peak oxygen consumption (VO_2peak_) and peak power output (watts or W) derived from a maximal, incremental exercise test on an electronically-braked, computer-driven cycle ergometer (Lode BV, Groningen, The Netherlands) and a calibrated open-circuit spirometry system (TrueOne, Parvo Medics, Sandy, UT) for analyzing expired gases [55, 56]. The incremental exercise test initially involves a brief, 3-min warm-up at 0 W. The initial work rate for the incremental exercise test is 0 W, and the work rate continuously increases at a rate of 15 W/min (0.25 W/sec) until participants reach maximal exertion defined as volitional fatigue. Oxygen consumption (VO_2_), respiratory exchange ratio (RER), and W are measured continuously by the open-circuit spirometry system and expressed as 20-s averages. Heart rate (HR) is displayed using a Polar HR monitor (Polar Electro Oy, Finland), and HR and rating of perceived exertion (RPE) are recorded every minute. VO_2peak_ is expressed in ml kg^−1^ min^−1^ and peak power output is expressed in W based on the highest recorded 20-s values when two or more of the following criteria are satisfied: (1) VO_2_ plateau with increasing W; (2) RER ≥ 1.10; (3) peak HR within 10 beats per minute of age-predicted maximum (i.e., ~ 1 SD); or (4) peak RPE ≥ 17 [55, 56].

#### Muscle strength

Bilateral, isometric knee extensor (KE) and knee flexor (KF) peak torque will be measured using an isokinetic dynamometer (Biodex System 3 Dynamometer, Shirley, NY) [51, 57]. Participants will be seated on the dynamometer consistent with the manufacturer's recommendations. Isometric torque will be assessed at 3 joint angles of 45°, 60° and 75°. Per joint angle, participants perform three, 5-s maximal knee extensions and one, 5-s maximal knee flexion. There is a rest period of 5-s between contractions within a set, and the rest period is 1 min between sets. The highest recorded peak torque for the stronger leg, regardless of joint angle, represents KE and KF isometric strength (N·m) [51, 57].

### Random assignment

After collection of baseline data, participants will be randomly assigned into either the exercise training condition or the control condition using a computerized process based on a random numbers sequence, and group allocation will be concealed. Participants will not be informed directly that the exercise training condition represents the experimental treatment condition and the stretching condition (i.e., attention and social contact control condition) represents the control condition, as both conditions are based on guidelines and likely have benefits in MS. To do this, the study will be advertised as comparing two different exercise approaches for managing consequences of MS and improving health indicators among persons with MS. We will measure treatment credibility after the first assigned treatment session using an adaptation of the Reaction to Treatment Questionnaire (RTQ) [58].

### Intervention condition – home-based aerobic and resistance exercise training

**Fig. 1:**
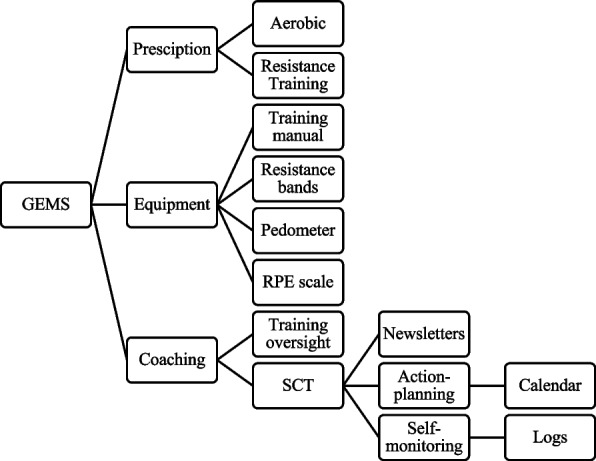
Outline of the Guideline for Exercise in Multiple Sclerosis (GEMS) program

The proposed trial will deliver the Guidelines for Exercise in MS (GEMS) program, as fully described in our previous research [18–22], within a remotely coached/guided, home-based setting using telerehabilitation (i.e., Zoom). The schematic of the main program components is provided in Fig. 1 and the components are summarized in Table 2. The intervention condition consists of six main components: (1) three different progressive trajectories of aerobic/resistance exercise prescriptions for individualization (Orange, Blue, and Green; Table 3) that are based on current guidelines for adults with MS who have mild-to-moderate disability (i.e., defined as EDSS 0–7) [23, 24], (2) appropriate exercise equipment including a CW-300 pedometer (NEW-LIFESTYLES, INC., Lee’s Summit, MO) and set of elastic resistance bands (Black Mountain Products, McHenry, IL), (3) one-on-one coaching, (4) action-planning via calendars, (5) log books for self-monitoring, and (6) SCT-based newsletters. Of note, the current exercise guidelines specify 30 + minutes of moderate-intensity aerobic exercise 3 time per week and resistance training targeting major muscle groups 3 times per week [23, 24]. Walking is the aerobic exercise modality based on it being the most commonly reported mode of exercise among people with mild MS [59] and the intensity walking is controlled based on a step rate of 100 steps per minute as this corresponds with moderate-intensity exercise in persons with MS [60]. The resistance training stimulus consists of 1–2 sets involving 10–15 repetitions of 5–10 exercises that target the lower body, upper body, and core muscle groups. The specific lower body exercises are the chair raise, calf raise, knee flexion, knee extension, and the lunge; the specific upper body resistance exercises are the shoulder row, shoulder raise, elbow flexion, and elbow extension; and the specific core exercise is the abdominal curl. The one-on-one coaching (i.e. weeks 1, 2, 3, 4, 5, 7, 11, and 15) focuses on three main components: (1) exercise training guidance and oversight, (2) discussion of the behavioral strategies of action planning and self-monitoring, and (3) presentation and discussion of newsletters based on SCT constructs (i.e., outcome expectations, self-monitoring, goal-setting, self-efficacy, barriers, and facilitators) for optimizing adherence and compliance (Table 4) [18–22]. We further provide all participants with an NMSS educational packet “Minimizing your risk for falls: A guide for people with MS”, and a study-specific instruction sheet on fall prevention. Participants are instructed to document any falls and other concerns or adverse events in the exercise adherence log and report these during one-on-one coaching, and all adverse events will be documented and reported per UIC IRB guidelines.

**Table 2:** Summary of Guidelines for Exercise in Multiple Sclerosis program features

**Feature**	**Description**
Prescription	Guidelines for Exercise in MS
	Progressive
	Individualized
Frequency	3 times per week
Exercise Session Duration	Approximately 1 h
Exercise Session Intensity	Moderate
	100 steps/min (pedometer)
	Rate of Perceived Exertion (RPE) between 11–13
Intervention Length	16 weeks
Meeting with Coach	
Weeks 1–4	Weekly
Weeks 5–8	Bi-weekly
Weeks 9–16	Monthly
Setting	Home
Supervision	
Who	Trained personnel
Mode	Remote, telecoaching
Exercise Modes	
Aerobic	Walking
Resistance	Bands, body weight
Materials Provided	Program Manual
	Newsletters
	Logbook
	Calendar
	Online Exercise Videos Accessible through QR Code
Equipment Provided	Pedometer
	Bands
Training Oversight	Zoom/Exercise Videos
Behavioral Intervention	Zoom
Intervention Safety	Fall Risk Assessment
	NMSS Resources
	Fall Prevention Instructions
	Zoom Oversight and AE/SAE Reporting

The table and its contents are adapted from our previous research using the same intervention [18–22]

**Table 3:** Progression of the 3 levels of individualization of the Guidelines for Exercise in Multiple Sclerosis program

Week	Orange		Blue		Green	
	Aerobic training	Resistance training	Aerobic training	Resistance training	Aerobic training	Resistance training
	Phase I					
1	10 min, ~ 1000 steps			1S, 10R, 5E		
2	10 min, ~ 1000 steps			1S, 12R, 5E		
	Phase II					
3	15 min, ~ 1500 steps	1S, 15R, 5E	15 min, ~ 1500 steps	1S, 12R, 5E	10 min, ~ 1000 steps	1S, 12R, 5E
4	20 min, ~ 2000 steps	2S, 10R, 5E	15 min, ~ 1500 steps	1S, 15R, 5E	15 min, ~ 1500 steps	1S, 12R, 5E
5	25 min, ~ 2500 steps	2S, 12R, 5E	20 min, ~ 2000 steps	2S, 10R, 5E	15 min, ~ 1500 steps	1S, 15R, 5E
6	30 min, ~ 3000 steps	2S, 15R, 5E	20 min, ~ 2000 steps	2S, 12R, 5E	20 min, ~ 2000 steps	2S, 10R, 5E
7	30 min, ~ 3000 steps	2S, 15R, 6E	25 min, ~ 2500 steps	2S, 15R, 5E	20 min, ~ 2000 steps	2S, 10R, 5E
8	30 min, ~ 3000 steps	2S, 15R, 6E	30 min, ~ 3000 steps	2S, 15R, 5E	25 min, ~ 2500 steps	2S, 12R, 5E
9	30 min, ~ 3000 steps	2S, 15R, 7E	30 min, ~ 3000 steps	2S, 15R, 6E	25 min, ~ 2500 steps	2S, 12R, 5E
10	30 min, ~ 3000 steps	2S, 15R, 7E	30 min, ~ 3000 steps	2S, 15R, 6E	30 min, ~ 3000 steps	2S, 15R, 5E
11	30 min, ~ 3000 steps	2S, 15R, 8E	30 min, ~ 3000 steps	2S, 15R, 7E	30 min, ~ 3000 steps	2S, 15R, 6E
12	30 min, ~ 3000 steps	2S, 15R, 8E	30 min, ~ 3000 steps	2S, 15R, 8E	30 min, ~ 3000 steps	2S, 15R, 6E
13	30 min, ~ 3000 steps	2S, 15R, 9E	30 min, ~ 3000 steps	2S, 15R, 8E	30 min, ~ 3000 steps	2S, 15R, 7E
14	30 min, ~ 3000 steps	2S, 15R, 9E	30 min, ~ 3000 steps	2S, 15R, 9E	30 min, ~ 3000 steps	2S, 15R, 8E
15	30 min, ~ 3000 steps	2S, 15R, 10E	30 min, ~ 3000 steps	2S, 15R, 10E	30 min, ~ 3000 steps	2S, 15R, 9E
16	30 min, ~ 3000 steps	2S, 15R, 10E	30 min, ~ 3000 steps	2S, 15R, 10E	30 min, ~ 3000 steps	2S, 15R, 10E

*S* number of sets, *R* number of repetitions, *E* number of exercises

The table and its contents are adapted from our previous research using the same intervention [18–22]

**Table 4:** Behavioral coaching session content

Week 1 Introduction to program	*Tele/Video-chat 1:* Clarification of materials received and initial questions; Explanation of program; Planning exercise schedule; Using the log-book; *Newsletter 1;* Exercise expectations; Exercise outcomes; Importance of this knowledge
Week 2 Outcome expectations	*Tele/Video-chat 2:* Compliance with program; Using the manual and log-book; Identifying personal outcomes
Week 3 Choosing a program	*Tele/Video-chat 3;* Compliance with program; Comparison of orange, blue and green programs; Choosing a program; *Newsletter 2;* Self-monitoring defined; Benefits of self-monitoring; Importance of this knowledge
Week 4 Self-monitoring	*Tele/Video-chat 4:* Compliance with program; Using your pedometer; Understanding exercise intensity
Week 5 Goal-setting	*Tele/Video-chat 5:* Compliance with program; Setting SMAART goals; Performing resistance training exercises correctly; Tracking progress; *Newsletter3;* Specific, measurable, adjustable, action-oriented, realistic, and time-limited exercise related goals defined; Importance of this knowledge
Week 7 Self-efficacy	*Tele/Video-chat 6:* Finding your self-confidence; What to do when you feel like quitting; Involving family; *Newsletter 4;* Self-efficacy defined; Experiencing success, choosing role models, accepting encouragement & managing physical and emotional responses; Reminder that program is specific for persons with MS
Week 11 Overcoming Barriers	*Tele/Video-chat 7:* Identifying your barriers; Making plans to overcome obstacles; Dealing with MS symptoms; *Newsletter 5;* Exercise barriers defined; Common barriers (facilities, social & symptoms); Strategies to overcome barriers
Week 15 Identifying facilitators	*Tele/Video-chat 8:* How to keep going on your own; Making adjustments as needed; Setting future goals; *Newsletter 6;* Exercise facilitators defined; Common facilitators (having a goal, enjoyment, social support, knowledge); Using facilitators long term

The table and its contents are adapted from our previous research using the same intervention [18–22]

### Control condition – home-based stretching and flexibility program

This program has been described in our previous research [22] and was developed based on a RCT of exercise training for improving mobility in MS [51] and two RCTs of exercise training for cognitive dysfunction in MS [61, 62]. The program itself has identical components as the GEMS program for aerobic and resistance exercise training, but focuses on stretching for improving flexibility and range of motion as important components of fitness. The program itself is based on *Stretching for People with MS: An Illustrated Manuel* from the National MS Society (Table 5), as this is MS specific and enhances the credibility of the control condition. Participants will be provided with a yoga pad (i.e., exercise equipment) and a manual, log-book, calendar, and prescription for the stretching program. This program includes newsletters focusing on SCT for behavior change, and video-chats with behavioral coaches that provide motivation and social accountability. The video-chats occur on the same timeline and frequency as the GEMS exercise training program in the intervention condition, but focus on the SCT constructs applied for stretching. We further monitor safety and compliance as done in the intervention condition, and provide resources and instruction on safety. Importantly, this condition accounts for the possible influences of social-contact and attention associated with the GEMS program on the study outcomes, and this represents a major advancement over waitlist control and standard of care conditions in previous RCTs of exercise training and depression in MS [10, 15–17].

**Table 5:** Progression of the 3 levels of individualization of the stretching prescription

Week	Orange	Blue		Green
	Phase I			
1	10 min, 5 categories		2S, 2E, 15 s	
2	10 min, 5 categories		2S, 2E, 15 s	
	Phase II			
3	15 min, 5 categories 2S, 3E, 25 s	15 min, 5 categories 2S, 3E, 20 s		10 min, 5 categories 2S, 2E, 15 s
4	20 min, 5 categories 3S, 3E, 30 s	15 min, 5 categories 2S, 3E, 20 s		15 min, 5 categories 2S, 3E, 20 s
5	25 min, 5 categories 3S, 4E, 45 s	20 min, 5 categories 2S, 3E, 30 s		15 min, 5 categories 2S, 3E, 20 s
6	30 min, 5 categories 4S, 4E, 60 s	20 min, 5 categories 3S, 3E, 30 s		20 min, 5 categories 3S, 3E, 30 s
7	30 min, 6 categories 4S, 4E, 60 s	25 min, 6 categories 3S, 3E, 45 s		20 min, 6 categories 3S, 3E, 30 s
8	30 min, 6 categories 4S, 4E, 60 s	30 min, 6 categories 4S, 4E, 60 s		25 min, 6 categories 4S, 3E, 45 s
9	30 min, 7 categories 4S, 4E, 60 s	30 min, 7 categories 4S, 4E, 60 s		25 min, 7 categories 4S, 3E, 45 s
10	30 min, 7 categories 4S, 4E, 60 s	30 min, 7 categories 4S, 4E, 60 s		30 min, 7 categories 4S, 4E, 60 s
11	30 min, 8 categories 4S, 4E, 60 s	30 min, 8 categories 4S, 4E, 60 s		30 min, 8 categories 4S, 4E, 60 s
12	30 min, 8 categories 4S, 4E, 60 s	30 min, 8 categories 4S, 4E, 60 s		30 min, 8 categories 4S, 4E, 60 s
13	30 min, 8 categories 4S, 4E, 60 s	30 min, 8 categories 4S, 4E, 60 s		30 min, 8 categories 4S, 4E, 60 s
14	30 min, 8 categories 4S, 4E, 60 s	30 min, 8 categories 4S, 4E, 60 s		30 min, 8 categories 4S, 4E, 60 s
15	30 min, 8 categories 4S, 4E, 60 s	30 min, 8 categories 4S, 4E, 60 s		30 min, 8 categories 4S, 4E, 60 s
16	30 min, 8 categories 4S, 4E, 60 s	30 min, 8 categories 4S, 4E, 60 s		30 min, 8 categories 4S, 4E, 60 s

*S* number of sets, *E* number of exercises

The table and its contents are adapted from our previous research using the same control condition [22]

### Procedure

The study procedure is administered by a project coordinator with oversight by the PI and Co-Is, and monitored through a fidelity monitoring plan (Table 6). As done in our previous research [18–22], the project coordinator will contact interested participants via telephone, describe the study and its requirements, and then conduct the screening for inclusion/exclusion criteria. The project coordinator will then distribute the informed consent document electronically among participants who meet inclusion criteria further information about the study. This will be followed by a telephone call that ensures participants received the document and understand the study and research procedures. The project coordinator will further work with participants in obtaining physician approval for participation and verification of MS diagnosis as a final step in enrollment.

**Table 6:** Overview of fidelity monitoring plan

		Areas of fidelity monitored				
Data source	Monitoring frequency	Study design	Provider training	Treatment delivery	Treatment receipt	Treatment enactment
Coaching call checklist	Monthly	** × **	** × **	** × **		
Coaching call logs	Monthly	** × **		** × **		
Auditing of coaching calls by expert	Weekly	** × **	** × **	** × **	** × **	
Behavioral resource bank within treatment group	Quarterly	** × **				
Review of participant exercise logbook	Weekly/ Monthly				** × **	** × **
Team meetings to discuss participant progress and protocol adherence	Weekly	** × **	** × **	** × **	** × **	** × **

The project coordinator will schedule baseline data collection, and provide written and verbal instructions regarding the baseline testing procedures. The project coordinator will send the participant document with directions and parking information, and contact the participant electronically and through telephone 24-h before the appointment as a reminder. Upon arrival, the project coordinator will review the study procedures with the participant, obtain written informed consent, and then initiate the baseline data collection.

The baseline data collection will be undertaken by treatment-blinded researchers who will start with a PAR-Q for ensuring safety and then administer measures of depression severity (i.e., primary outcomes) followed by the BICAMS and measures of fatigue and HRQOL (i.e., secondary outcomes). The participant will then undertake the maximal exercise test and muscle strength testing with a 15-min break between the measures of fitness. The competition of those measures will take ~ 120 min based on our previous experiences.

The treatment-blinded researchers will provide the participant with a packet containing an accelerometer along with GLTEQ. This packet will include instructions regarding the importance of wearing the accelerometer as instructed every day during the seven-day period, and provide a pre-stamped and pre-addressed envelope for return postal service. The participants will wear the accelerometer for a seven-day period and then complete the GLTEQ. The project coordinator will send brief, scripted e-mails for reminding participants about wearing the accelerometer in the middle of the seven-day period. This will be followed by a telephone call verifying that participants wore the accelerometer daily during the seven-day period and returned it along with the GLTEQ through the United States Postal Service.

Of note, demographic and disease-related characteristics will be collected from participant interviews and verification forms from the treating Neurologist, respectively. The patients will further provide a list of current medications and ongoing treatments for MDD and other symptoms of MS.

Once the baseline assessment is completed, participants will be randomly assigned into either the intervention or control conditions using a random numbers sequence with concealed allocation. The project coordinator will receive information on allocation, record it in a database, and communicate the condition of assignment with the participant and behavioral coaches. Importantly, several strategies will be adopted for maintaining blinded conditions. The behavioral coaches and other study staff are located in a separate lab space from where the treatment-blinded researchers administer outcomes. The behavioral coaches will emphsize among participants the importance of not revealing what type of exercise is being undertaken when interactiong with outcome assessors. The study staff will remind participants about not revealing the type of exercise being undertaken before the outset of follow-up outcome assessments.

The intervention/control conditions will be delivered by behavioral coaches who are univolved in outcome assessments in 12, partially overlapping waves of ~ 12 participants per wave, and the conditions will be delivered across a 4-month period. This use of waves will afford additional time for behavioral coaching during the one-on-one chat sessions than if enrolling 146 in one recruitment wave. This should permit greater penetration of the study materials. Participants will be asked to contact the project coordinator via the dedicated toll-free telephone number or e-mail in the occurrence of an adverse event or any other problem; this information will further be collected during video chats with behavioral coaches. The project coordinator will administer the PHQ-9 on the same weeks as the behavioral chats for ongoing monitoring of the mood status of participants.

The participants will complete the same measurement procedures immediately (i.e., immediate follow-up; 4 months) and 4-months (i.e., long-term follow-up; 8 months) after initiating the intervention/control conditions. There will be no behavioral coaching session during the long-term follow-up period for examining sustainability.

Participants will receive $100 USD remuneration for completing the measures per assessment period, including baseline, for a total of $300 USD. We will collect formative feedback using a Qualtrics survey for identifying opportunities for intervention improvement and refinement; this will be undertaken by participants after completion of the study.

### Data analyses

#### Overview

The data analyses will be overseen by a biostatistician and follow intent-to-treat (ITT) principles (i.e., include all persons regardless of dropout). We will perform exploratory data analyses only among those who complete immediate and long-term follow-up testing (i.e., completer’s or per protocol analysis). We will check the data for errors and outliers, and lock the data set before analyses. The analytic plan will account for potential confounders of the intervention effect on the outcomes. The confounders may include MS duration, BMI, age, sex, disease-modifying therapy, and relapse rate. We will include any of those variables and others that differ between conditions as covariates in the following analyses.

#### Data analysis – aim 1

The first analysis tests the hypothesis that those who are randomly assigned into the intervention condition (i.e., exercise training) will demonstrate (a) reductions from baseline in depression severity that (b) are sustained over 4-months of follow-up compared with those in the control condition (i.e., stretching). The hypothesis will be tested using a linear mixed model in JMP Pro 16.0. The linear mixed model will include condition and time as fixed effects, and subject nested within condition as a random effect using unbounded variance components and the REML method (https://www.jmp.com/content/dam/jmp/documents/en/academic/learning-library/08-repeated-measures-analysis-(mixed-model).pdf). The hypothesized interaction term will be decomposed with follow-up tests, and differences in comparison of mean scores will be expressed as Cohen’s d with standard guidelines for interpretation. The final models for PHQ-9 and HDRS-17 scores will be adjusted for covariates. The overall Type I error will be controlled based on an adjustment of alpha (two-tailed *α* = 0.025) given the two primary outcomes in Aim 1.

#### Data analysis – aim 2

The second set of analyses test the hypotheses that those who are randomly assigned into the intervention condition (i.e., exercise training) will report (a) improvements from baseline in fatigue, cognitive performance, and QOL that (b) are sustained over 4-months of follow-up compared with those in the control condition (i.e., stretching). Those hypotheses will be tested with the same modeling approach described for Aim 1. The overall Type I error will be controlled using a step-down procedure testing first fatigue, followed by domains of cognitive performance (SDMT, CVLT-II, and then BVMT-R), and lastly HRQOL [63].

#### Data analysis – aim 3

The third set of analyses test the hypotheses that those who are randomly assigned into the intervention condition (i.e., exercise training) will report (a) improvements from baseline in exercise behavior, free-living PA, and aerobic capacity and muscle strength that (b) are sustained over 4-months of follow-up compared with those in the control condition (i.e., stretching). Those hypotheses will be tested with the same modeling approach described for Aim 1. The overall Type I error will be controlled using a step-down procedure testing first exercise behavior and free-living PA, followed by aerobic capacity and muscle strength as outcomes [63].

### Current trial status

As of February 13, 2024 and reported in our quarterly report for the funder, we have enrolled 18 persons into the trial, and these persons have been equally randomized into the intervention and control conditions (9 per condition). There were 6 other persons scheduled for baseline testing and ready for randomization.

## Discussion

We are proposing a Phase-II RCT of exercise training for treating depression severity in persons with MS who have MDD. If successful based on statistically significant and clinically meaningful improvements in depression symptom outcomes (e.g., ½ SD improvement for exercise compared with control) [64] as well as retention exceeding 20% (primary decision rules), we will proceed with the design of a Phase-III clinical trial of exercise training compared with CBT alone and combined with exercise training for treating depression severity in persons with MS who have MDD. We propose the addition of CBT as it has been considered a “possibly efficacious” treatment for depression in MS [7, 8] and can be delivered remotely [9]. This is a logical next step, as the data gathered herein would power such a clinical trial and provide necessary experiences for a presumed larger trial. We further have experience in the conduct of Phase-III trials of exercise and physical activity in MS [21, 65], and our ongoing PCORI trial provides a benchmark for conducting a Phase-III clinical trial of exercise training compared with CBT for managing depression severity in persons with MS who have MDD. Such a Phase-III clinical trial would provide definitive evidence for transition into clinical care and practice of persons with MS who have MDD, perhaps serve as a benchmark for studying exercise training as a treatment of other outcomes in persons with MS – this is a major stumbling block in all MS research involving exercise training [66], including depressive symptoms in MDD [10].

We may experience problems with the participants adhering with the intervention and control conditions based on the lack of interest/pleasure in activities, sadness, tiredness/fatigue, or physical problems (e.g., pain) as part of MDD. We are minimizing this by using SCT-based content and strategies and 1:1 remote behavioral coaching for maximizing adherence with both conditions. We further are managing this by enrolling a smaller number of persons (n ~ 12) over 12, partially overlapping recruitment waves (i.e., 12 waves of ~ 12 participants per wave), and thereby having the behavior coaches devote a greater amount of time with the participants during the one-on-one chat sessions. This should permit greater penetration of the study materials and a larger change in behavior for both conditions. The power analysis was based on meta-analyses for the effect of exercise interventions on depressive symptoms in samples that were not prescreened for MDD, and the preliminary data might not represent the treatment effect for those with MDD. Of note, our secondary analysis of previously published data suggested that effect of a physical activity intervention on depressive symptoms was stronger in those with elevated scores [67], and this would suggest that our power analysis and sample size should be appropriate for detecting an intervention effect on depression in those with MDD. There may be some attrition during the 4-month follow-up period wherein there is no planned coaching/contact, but this has been minimal in our previous [68] and ongoing [21] trials using the sample general approach; this is expected as the conditions are designed around teaching people skills, techniques, and strategies for sustainable behavior change.

If successful, the proposed study will provide the first and only Class I evidence for a home-based exercise training program as a treatment of depression in persons with MS who have MDD. This is critical as exercise training would likely have secondary effects on symptoms, cognition, and HRQOL, and provide a powerful, behavioral approach for managing the many negative outcomes of MDD in MS. The program in the proposed research is accessible and scalable for broad-scale treatment of depression in MS, and provides the potential for integration in the clinical management of this disease.

### Supplementary Information

**Supplementary Material 1.**

## Data Availability

No datasets were generated or analysed during the current study.

## Abbreviations

AEs: Adverse Events
BICAMS: Brief International Cognitive Assessment for MS
BVMT-R: Brief Visuospatial Memory Test-Revised
CVLT-II: California Verbal Learning Test-II
CNS: Central Nervous System
CBT: Cognitive Behavioral Therapy
Co-Is: Co-Investigators
CER: Comparative Effectiveness Research
DSM-V: Diagnostic Manual of Mental Disorders V
DMTs: Disease Modifying Therapies
ES: Effect Size
ENRL: Exercise Neuroscience Research Laboratory
FSS: Fatigue Severity Scale
GEMS: Guidelines for Exercise in Multiple Sclerosis
GLTEQ: Godin Leisure-Time Exercise Questionnaire
HDRS-17: Hamilton Depression Rating Scale
HCS: Health Contribution Score
HRQOL: Health-Related Quality of Life
HR: Heart Rate
IRB: Institutional Review Board
KE: Knee Extensor
KF: Knee Flexor
MDD: Major Depressive Disorder
MFIS: Modified Fatigue Impact Scale
MS: Multiple Sclerosis
MSIS-29: Multiple Sclerosis Impact Scale
NMSS: National MS Society
VO_2_: Oxygen Consumption
PDDS: Patient-Determined Disease Steps
PHQ-9: Patient Health Questionnaire-9
VO_2peak_: Peak Oxygen Consumption
W: Peak Power Output
PA: Physical Activity
PAR-Q: Physical Activity Readiness Questionnaire
PI: Principal Investigator
RCT: Randomized Controlled Trial
RPE: Rating of Perceived Exertion
RRMS: Relapsing-Remitting MS
RER: Respiratory Exchange Ratio
SAEs: Serious Adverse Events
SCT: Social-Cognitive Theory
SDMT: Symbol Digit Modalities Test
TICS-M: Telephone Interview for Cognitive Status
USD: United States Dollar
